# Live birth rates and perinatal outcomes when all embryos are frozen compared with conventional fresh and frozen embryo transfer: a cohort study of 337,148 in vitro fertilisation cycles

**DOI:** 10.1186/s12916-019-1429-z

**Published:** 2019-11-13

**Authors:** Andrew D. A. C. Smith, Kate Tilling, Deborah A. Lawlor, Scott M. Nelson

**Affiliations:** 1 Applied Statistics Group, University of the West of England, Bristol, BS16 1QY UK; 2 MRC Integrative Epidemiology Unit at the University of Bristol, Bristol, BS8 2BN UK; 3 Population Health Science, Bristol Medical School, Bristol, UK; 4 NIHR Bristol Biomedical Research Centre, Bristol, UK; 5 School of Medicine, New Lister Building, Glasgow Royal Infirmary, University of Glasgow, Glasgow, G31 2ER UK

**Keywords:** IVF, Freeze all, Perinatal outcome, Frozen embryo transfer

## Abstract

**Background:**

It is not known whether segmentation of an in vitro fertilisation (IVF) cycle, with freezing of all embryos prior to transfer, increases the chance of a live birth after all embryos are transferred.

**Methods:**

In a prospective study of UK Human Fertilisation and Embryology Authority data, we investigated the impact of segmentation, compared with initial fresh embryo followed by frozen embryo transfers, on live birth rate and perinatal outcomes. We used generalised linear models to assess the effect of segmentation in the whole cohort, with additional analyses within women who had experienced both segmentation and non-segmentation. We compared rates of live birth, low birthweight (LBW < 2.5 kg), preterm birth (< 37 weeks), macrosomia (> 4 kg), small for gestational age (SGA < 10th centile), and large for gestational age (LGA > 90th centile) for a given ovarian stimulation cycle accounting for all embryo transfers.

**Results:**

We assessed 202,968 women undergoing 337,148 ovarian stimulation cycles and 399,896 embryo transfer procedures. Live birth rates were similar in unadjusted analyses for segmented and non-segmented cycles (rate ratio 1.05, 95% CI 1.02–1.08) but lower in segmented cycles when adjusted for age, cycle number, cause of infertility, and ovarian response (rate ratio 0.80, 95% CI 0.78–0.83). Segmented cycles were associated with increased risk of macrosomia (adjusted risk ratio 1.72, 95% CI 1.55–1.92) and LGA (1.51, 1.38–1.66) but lower risk of LBW (0.71, 0.65–0.78) and SGA (0.64, 0.56–0.72). With adjustment for blastocyst/cleavage-stage embryo transfer in those with data on this (329,621 cycles), results were not notably changed. Similar results were observed comparing segmented to non-segmented within 3261 women who had both and when analyses were repeated excluding multiple embryo cycles and multiple pregnancies. When analyses were restricted to women with a single embryo transfer, the transfer of a frozen-thawed embryo in a segmented cycles was no longer associated with a lower risk of LBW (0.97, 0.71–1.33) or SGA (0.84, 0.61–1.15), but the risk of macrosomia (1.74, 1.39–2.20) and LGA (1.49, 1.20–1.86) persisted. When the analyses for perinatal outcomes were further restricted to solely frozen embryo transfers, there was no strong statistical evidence for associations.

**Conclusions:**

Widespread application of segmentation and freezing of all embryos to unselected patient populations may be associated with lower cumulative live birth rates and should be restricted to those with a clinical indication.

## Background

In vitro fertilisation (IVF) commonly involves ovarian stimulation to produce a number of oocytes, followed by fertilisation of these oocytes and fresh embryo transfer, with surplus embryos frozen for transfer in subsequent menstrual cycles. However, recent advances have led some to promote stimulation of the ovaries to produce an excess of oocytes and then freezing all embryos before transferring them to the woman at a later time (known as segmentation of the IVF cycle) [1–3]. As couples undertaking assisted conception wish to maximise the chance of having a healthy baby, consideration of whether segmentation will improve the live birth rate and reduce adverse perinatal outcomes is critical.

The evidence suggesting segmentation may increase live birth rates is largely based on observational studies, with limited confounder adjustment, that report rates in frozen embryo compared with fresh embryo transfers, rather than explicitly comparing segmentation with non-segmentation [4–7]. Registry studies have also not considered that the underlying patient prognosis of women with fresh and frozen embryos transfers may differ, with women experiencing a frozen embryo transfer more likely to have a higher oocyte yield, a larger cohort of embryos to select from, and a blastocyst transfer. Furthermore, these studies with few exceptions [8, 9] have not generally reported the live birth rate accounting for all embryos transferred during an ovarian stimulation cycle, but primarily focused on maternal and perinatal outcomes per embryo transfer. They find some evidence for reduced risk of preterm birth and lower birthweight with segmentation, but higher risk of hypertensive disorders of pregnancy, large for gestational age (LGA), and neonatal and infant mortality.

Of 6 randomised trials comparing segmentation to non-segmentation, 2 were in highly selected clinical populations, with 1 only including women with polycystic ovarian morphology (131 women) [10], and the other only including women with polycystic ovarian syndrome (1508 women) [11]. A third trial only included women with an anticipated normal ovarian response in analyses (101 of the 411 women initially randomised) [12]. While these studies suggested a benefit of segmentation, their select populations may limit the generalisability of their findings to the majority of women receiving IVF. A trial with wider inclusion criteria and that included all randomised women in analyses (782 women) did not observe an improvement in live birth rates or fewer complications related to ovarian stimulation with segmentation [13]. Similarly, a larger trial (2157 women) where a normal response to ovarian stimulation was anticipated, there was no improvement in live birth rates with segmentation of the cycle. A more recent trial of good prognosis patients (1650 women) using contemporary laboratory techniques and blastocyst culture did observe an improvement if the initial embryo transfer was frozen [14]. Only 2 of the 6 trials have examined cumulative live birth rates, and despite these, initial apparent improvements for the first transfer have not found a sustained improvement in cumulative live birth rates between segmented and non-segmented cycles [11, 14], consistent with a recent observational study [15].

The aim of this study was to determine whether segmentation of the IVF cycle improves the likelihood of a healthy live birth per IVF cycle initiated.

## Methods

### Source of data

We analysed an anonymised database provided by the Human Fertilisation and Embryology Authority (HFEA), who have a statutory duty to collect data on all in vitro fertilisation treatment in the UK. By law, clinicians provide details of patients, their treatment, and its outcome. The HFEA provided data between 2003 and 2013 inclusive, including linkage of individual embryo transfers to ovarian stimulation. The HFEA provided ethical approval for this study.

**Fig. 1:**
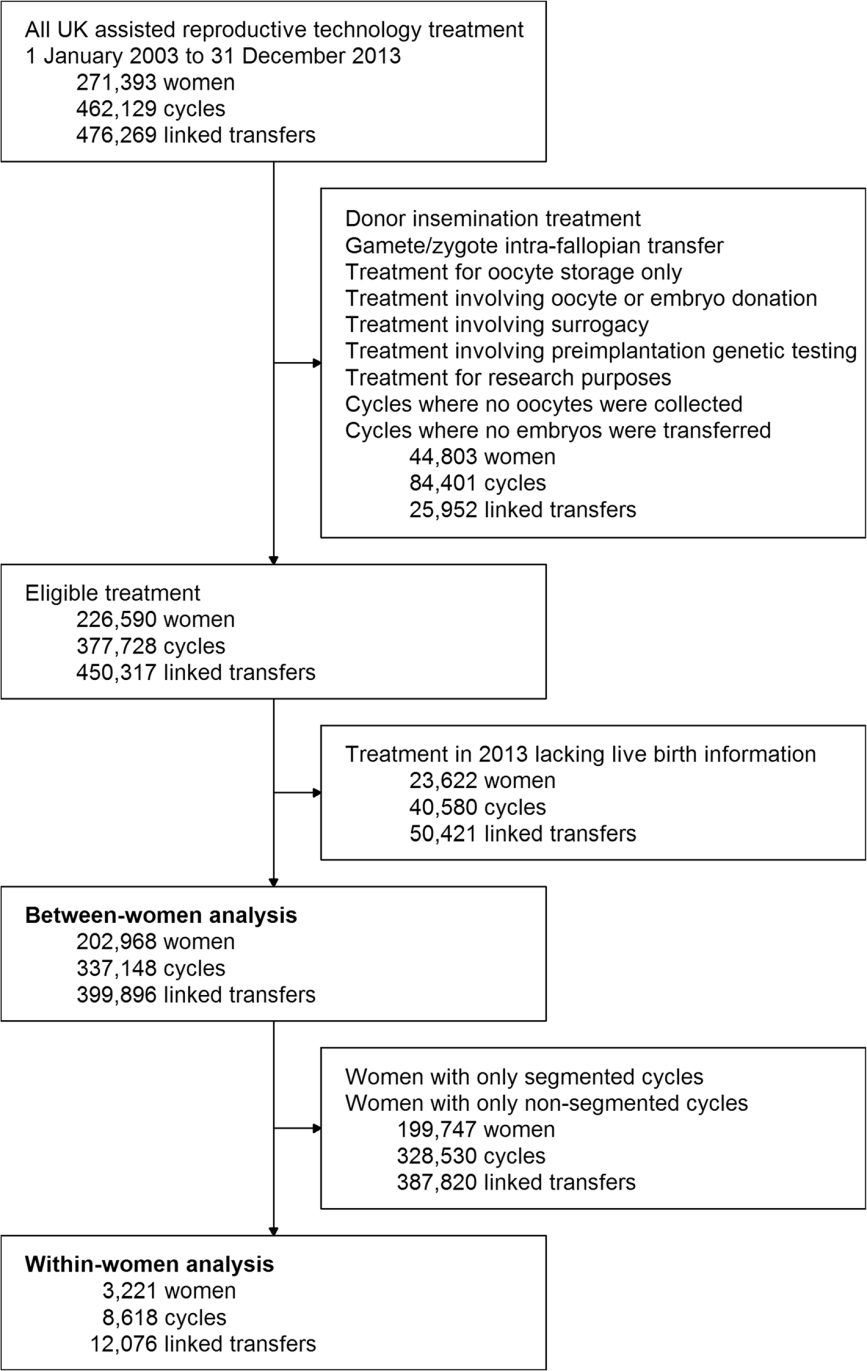
Formation of the analysis cohorts

We excluded all non-IVF treatments (i.e. donor insemination (DI) and gamete/zygote intrafallopian transfer (GIFT/ZIFT)) as well as treatment involving oocyte donation, embryo donation, preimplantation genetic testing, or surrogacy. Ovarian stimulation for the sole purpose of storing oocytes was also excluded, as was the treatment for the sole purpose of research. Cycles where no oocytes were collected and therefore neither segmented nor non-segmented cycles could take place were excluded. Due to a lack of data regarding live birth and perinatal outcomes in those treated in 2013, we also excluded treatment from this year. Following these planned exclusions, the analysis cohort included 202,968 women undergoing 337,148 ovarian stimulation cycles and 399,896 embryo transfer procedures (Fig. 1).

### Definitions

A cycle of IVF was defined as a planned ovarian stimulation followed by transfer of all fresh and/or frozen embryos. Live birth was defined as at least one infant born after 24 weeks of gestation and surviving for 1 month. Our primary outcome measure was the cumulative live birth rate within the IVF cycle, defined as the probability of at least one live birth in a given ovarian stimulation cycle, i.e. the live birth rate from all fresh embryo and/or frozen embryo transfers following ovarian stimulation. We defined a segmented cycle as ovarian stimulation followed by creation and freezing then thawing of embryos and transfer of frozen embryos only. A non-segmented cycle was defined as ovarian stimulation followed by creation and immediate transfer of one or more fresh embryo(s), with possible freezing, thawing, and transfer of frozen embryos.

Patient age in years, number of oocytes retrieved, and cause of infertility were reported by clinicians in the HFEA data. As the HFEA data linked treatment to individual women, we were able to ascertain the number, nature, and outcome of previous IVF cycles.

We defined preterm birth as gestational age less than 37 weeks; we also examined the associations with very preterm birth (less than 33 weeks). Birthweight was provided in 100-g increments and categorised as low birthweight (less than 2.5 kg) and very low birthweight (less than 1.5 kg). Macrosomia was defined as birthweight greater than 4.0 kg. Small for gestational age (SGA) and LGA were defined as below the 10th and above the 90th percentile, respectively, of UK sex and gestational age-standardised reference charts [16]. SGA and LGA could not be defined for 2025 and 585 livebirths, respectively, because the HFEA combined those with birthweight less than 1 kg into 1 category to ensure participant anonymity or because the gestational age was above the range of the reference charts. For multiple births, the earliest gestational age and lowest birthweight (or in the case of macrosomia and large for gestational age, highest birthweight) were used for categorisation. In cycles in which more than 1 embryo transfer procedure resulted in a live birth, only the first live birth was considered.

### Statistical methods

We compared cumulative live birth rates and perinatal outcomes within segmented and non-segmented cycles using a rate ratio calculated by a generalised linear model. To account for women who had more than one cycle, we calculated confidence intervals using robust standard errors that allowed for correlation within women [17]. We considered age at time of ovarian stimulation, cycle number, number of oocytes retrieved, cause of infertility, and developmental stage (whether blastocyst or cleavage-stage embryo was transferred) to be the potential confounders given their known associations with cumulative live birth rate within a cycle and likely influence on couple and clinician’s choice to segment a cycle [18]. Although in all adjusted models we used the individual woman’s age, we also considered the important age thresholds of 35 and 40 years, given the known monotonic decline in IVF success rates from age 35 and that many funders consider age 40 as a clinical threshold for the withdrawal of funding. Other potential confounders that are not in the HFEA dataset (and therefore not included in our models) are socio-economic position and associated characteristics such as body mass index and smoking.

To adjust for observed confounders, we included all eligible ovarian stimulation cycles and calculated unadjusted rate ratios and rate ratios adjusted for age, cycle number, number of oocytes retrieved, and cause of infertility. In our primary analyses, we did not adjust for the embryonic developmental stage because of differential missing data, but we did so in the additional analyses (see below). Details of how these confounders were categorised are provided in Additional file 1. Similar analyses of perinatal outcomes, restricted to those cycles with at least one live birth, were conducted. For cumulative live birth rates within cycles, we further calculated the rate ratios stratified by age categories, both unadjusted and adjusted for cycle number and the number of oocytes retrieved. To exclude the influence of multiple pregnancy on adverse perinatal outcomes, we repeated the analysis comparing the segmented and non-segmented cycles for those women with a singleton live birth, further adjusting by including a binary variable for previous IVF live birth. Furthermore, as adverse perinatal outcomes are associated with the number of embryos transferred [19], we investigated whether the effect of segmentation on perinatal outcomes could be mitigated by a single embryo transfer, by repeating the analysis restricted to live births following single embryo transfer. To assess whether our results were sensitive to changes over time in live birth rates, clinical practices, and embryo freezing technology, we repeated our analysis restricted to cycles initiated from 2011 onwards to cover the time period when vitrification of embryos was increasingly being adopted by IVF laboratories as the standard practice [20]. We also repeated our analyses restricted to the first embryo transfer in each cycle (i.e. the fresh embryo transfer in a non-segmented cycle and the first frozen embryo transfer in a segmented cycle) in order to examine the effect of segmentation on the transfer of the best quality embryo(s). We further repeated our analyses excluding live births after fresh embryo transfer, in order to examine the effect of segmentation on perinatal outcomes following frozen embryo transfer.

Blastocysts (as compared to cleavage-stage embryos) may be more likely to survive freezing (and hence be available for transfer), and this could influence clinician choices in relation to segmentation. There is also evidence that live birth rates are higher in cycles where blastocysts have been transferred [21]. Hence, developmental stage at freezing could confound the associations we have examined. Information on the developmental stage at freezing is not provided in the HFEA data, but the stage at transfer is provided. This information was differentially missing, with missing data on 37% of the segmented cycles and 1% of the non-segmented cycles (Table 1). As a result, we did not adjust for this variable in our main analyses. In additional complete case analyses, we did control for it in the association with live birth in those cycles with data on the developmental stage at transfer (5319 segmented and 324,302 non-segmented cycles) [22].

**Table 1:** Details of the analysis cohort of 337,148 cycles of IVF from 202,968 women

Number of cycles	Whole cohort, 337,148	Segmented cycles, 8393 (2.5%)	Non-segmented cycles, 328,755 (97.5%)
Patient age			
Less than 35 years	148,998 (44.2%)	5247 (62.5%)	143,751 (43.7%)
35–39 years	134,931 (40.0%)	2530 (30.1%)	132,401 (40.3%)
More than 39 years	53,219 (15.8%)	616 (7.3%)	52,603 (16.0%)
Cause of infertility (may be none or more than one)			
Male factor	142,982 (42.4%)	3402 (40.5%)	139,580 (42.5%)
Tubal	61,527 (18.2%)	1557 (18.6%)	59,970 (18.2%)
Ovulatory	40,696 (12.1%)	1814 (21.6%)	38,882 (11.8%)
Endometriosis	21,333 (6.3%)	448 (5.3%)	20,885 (6.4%)
Cycle number			
First cycle	173,776 (51.5%)	5625 (67.0%)	168,151 (51.1%)
Later cycle	163,372 (48.5%)	2768 (33.0%)	160,604 (48.9%)
Oocytes collected			
1–3	31,291 (9.3%)	172 (2.0%)	31,119 (9.5%)
4–9	141,837 (42.1%)	936 (11.2%)	140,901 (42.9%)
10–15	103,820 (30.8%)	1351 (16.1%)	102,469 (31.2%)
16+	60,200 (17.9%)	5934 (70.7%)	54,266 (16.5%)
Stage of the embryo at first transfer (% non-missing)			
Cleavage	270,919 (82.2%)	3742 (70.4%)	267,177 (82.4%)
Blastocyst	58,702 (17.8%)	1577 (29.6%)	57,125 (17.6%)
Missing (% all cycles)	7527 (2.2%)	3074 (36.7%)	4453 (1.4%)
At least one live birth following ovarian stimulation	105,174 (31.2%)	2748 (32.7%)	102,426 (31.2%)

In a second analysis, we attempted to adjust for both measured and unmeasured confounders by undertaking analyses within women who had undergone more than one treatment cycle and on whom at least one of the repeat cycles was segmented and one non-segmented. These analyses control for unmeasured confounders that do not change, or change relatively little (in comparison with differences between women), as women have repeat treatments. In these analyses, we assumed there was control for socio-economic position and reason for seeking IVF treatment, as these are likely to remain the same as a woman has repeat cycles, and that there was also some control for characteristics such as body mass index and smoking, which may change between treatment cycles within women, but are likely to be more similar within women across cycles than between different women. These analyses would be less likely to control for some factors that influence the choice of treatment, such as developmental stage, which may change between cycles. While this within-women analysis can better adjust for unmeasured confounders, it can introduce bias if the decision to switch strategies (change from segmentation to non-segmentation of the cycle or vice versa) is related to successful live birth. To deal with this in later cycles, we adjusted the rate ratios by including binary variables in the generalised linear models that recorded whether the woman had previously had a segmented cycle, whether she had a successful live birth in a previous cycle, and whether it was her last cycle. These adjusted rate ratios were stratified by the cycle number, restricted to no more than the third cycle to maintain at least 500 observations in each stratum.

There was a small amount of missing information on gestational age (0.6%), birthweight (2.3%), large for gestational age (3.4%), and SGA (4.2%); we undertook analyses for these outcomes on those with complete data (i.e. the vast majority of the cohort).

## Results

### Analysis 1: Between women including all eligible cycles

**Fig. 2:**
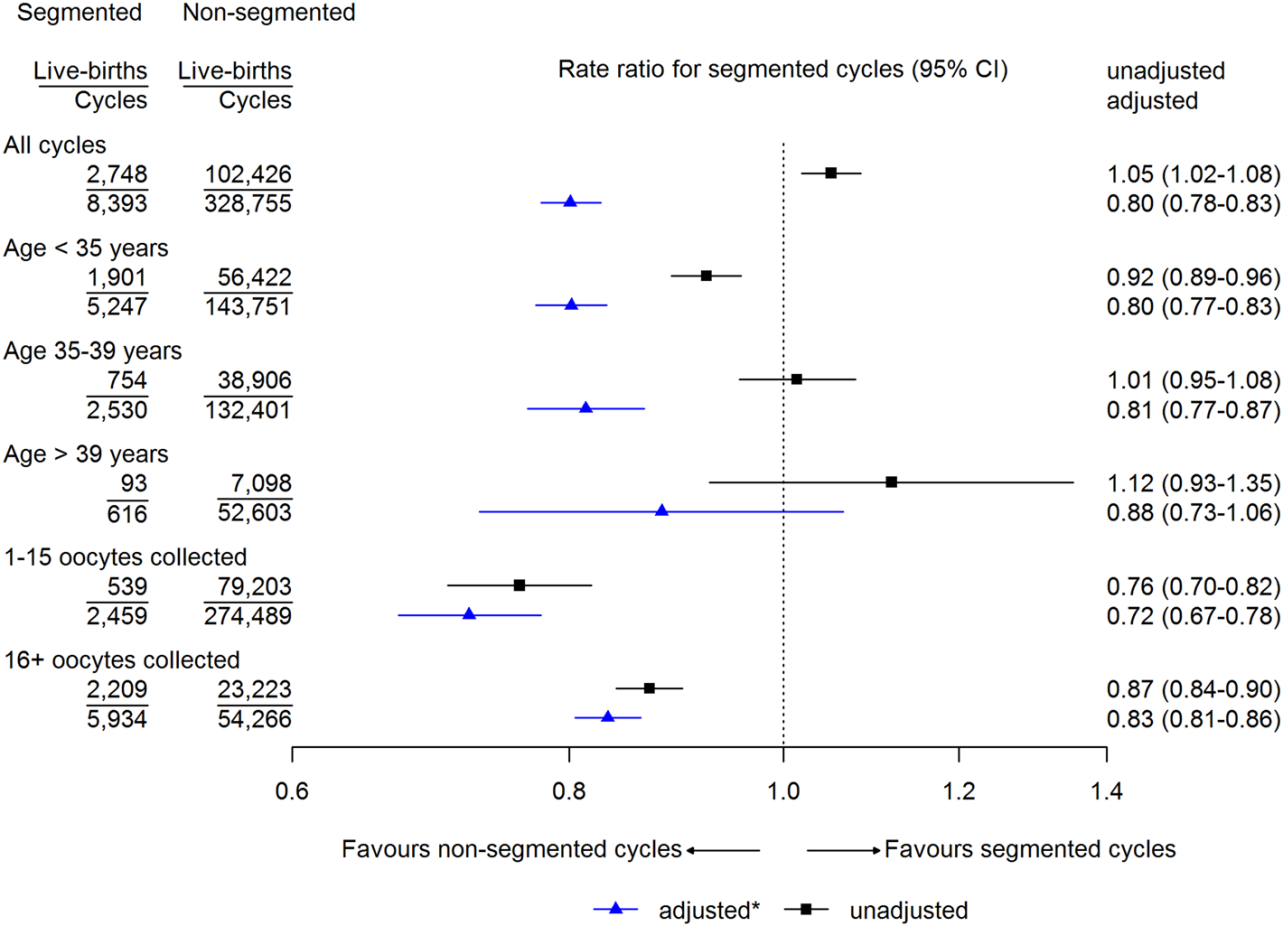
Live birth rate ratios for segmented cycles compared with non-segmented cycles, in 202,968 women undergoing 337,148 cycles of IVF. *Adjusted for age (where not stratified), cycle number, cause of infertility, and oocytes retrieved (where not stratified)

Of the 337,148 eligible cycles, 8393 (2.5%) were segmented (Table 1). Segmented cycles were more frequent in younger women, in first cycles, when more than 15 oocytes were retrieved, when the cause of infertility was ovulatory, and for blastocyst-stage embryos (Table 1). The unadjusted cumulative live birth rate within a cycle was slightly higher in segmented compared with non-segmented cycles (Fig. 2). However, in adjusted analyses, cumulative live birth rates within a cycle were lower in segmented cycles for all age groups and oocyte yields (Fig. 2).

**Fig. 3:**
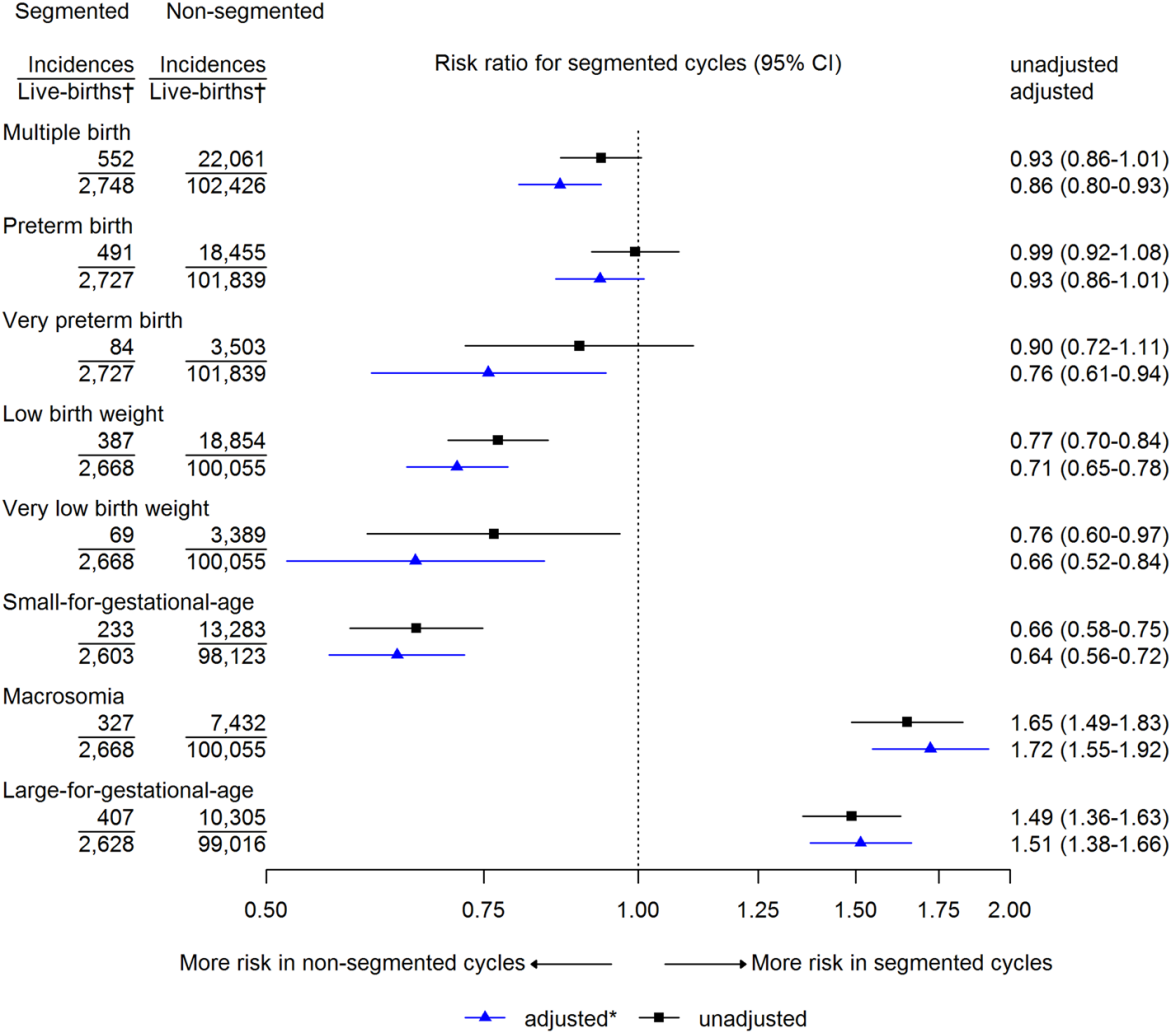
Risk ratios of perinatal outcomes following first live birth within a cycle, for segmented cycles compared with non-segmented cycles, in 105,174 live births from 202,968 women undergoing 337,148 cycles of IVF. *Adjusted for age, cycle number, cause of infertility, and oocytes retrieved. ^†^Information is non-missing

Multiple birth, low and very low birthweight, and SGA were less prevalent, and macrosomia and LGA were more prevalent, in segmented compared with non-segmented cycles (Fig. 3). These differences persisted after adjusting for age, cycle number, and oocytes retrieved. The different severities of preterm birth were similar in segmented and non-segmented cycles (Fig. 3). Similar results but with wider confidence intervals were observed when the analysis was restricted to the 82,561 singleton live births (Additional file 1: Figure S1). However, when the analysis was restricted to live births following single embryo transfer, there was no strong statistical evidence for the associations with low birthweight and SGA, but the increased prevalence of macrosomia and LGA with segmented cycles persisted (Additional file 1: Figure S2). When the analyses were repeated restricted to the most recent years of treatment (i.e. those from 2011 to 2013), the results were similar to those in the main analyses with all years of treatment included, but with wider confidence intervals (Additional file 1: Figure S3 and S4). When the analyses were stratified by stage of embryo development at transfer, the results were similar (Additional file 1: Figure S5).

When just the first embryo transfer in each cycle was considered, the unadjusted and adjusted live birth rates were lower for segmented cycles (Additional file 1: Figure S6), and the results for perinatal outcomes were similar to the main analysis (Additional file 1: Figure S7). When the analyses for perinatal outcomes were restricted to frozen embryo transfers, the sample size was restricted to 10,928 cycles, and there was no strong statistical evidence for associations (Additional file 1: Figure S8).

Of the 5645 segmented cycles that did not result in a live birth, 3657 (64.8%) used all stored embryos before commencing a new cycle or the end of the study period. Embryo storage took place in 95,477 (29.0%) of non-segmented cycles. Of the 49,975 non-segmented cycles with embryo storage that did not result in a live birth, 23,385 (46.8%) used all stored embryos before commencing a new cycle or the end of the study period.

### Analysis 2: Within women who had both segmented and non-segmented cycles

There were 3221 women who had both segmented and non-segmented cycles (Fig. 1). In these women, the cumulative live birth rate within a cycle was lower in segmented cycles than in non-segmented cycles. However, this difference may be biassed by the association of switching between treatments and the relationship of this to live birth. Furthermore, there were only 260 (8.0%) live births in the first cycle, reflecting the fact that restricting to women who had more than 1 cycle excludes women who had a live birth in their first cycle and then discontinued treatment. When we restricted analyses to the second and third cycles, with adjustment for previous segmentation, previous live birth, and whether it was the last cycle, evidence for lower live birth rates with segmented compared to non-segmented cycles remained, though the relatively small sample sizes means these results are less precisely estimated (wider confidence intervals) than the analyses presented above in all women (Table 2). We were unable to examine the perinatal outcomes due to the small number (155) of women who had both a live birth from a segmented cycle and a live birth from a non-segmented cycle.

**Table 2:** Comparison of live birth rate in segmented and non-segmented IVF cycles within 3221 women undergoing 8618 cycles of IVF, who had both segmented and non-segmented cycles

	Cycles (% cycles)	Live births* (live birth rate)	Rate ratio (95% CI)	Rate ratio, adjusted^†^ (95% CI)
All cycles				
Segmented	3361 (39.0%)	556 (16.5%)	0.70 (0.63, 0.76)	
Non-segmented	5357 (61.0%)	1250 (23.8%)	1	
First cycle				
Segmented	1720 (53.4%)	125 (7.3%)	0.81 (0.64, 1.02)	
Non-segmented	1501 (46.6%)	135 (9.0%)	1	
Second cycle				
Segmented	1150 (35.7%)	313 (27.2%)	0.85 (0.76, 0.95)	0.68 (0.50, 0.92)
Non-segmented	2071 (64.3%)	666 (32.2%)	1	1
Third cycle				
Segmented	340 (25.5%)	83 (24.4%)	0.83 (0.67, 1.02)	0.78 (0.59, 1.03)
Non-segmented	994 (74.5%)	294 (29.6%)	1	1

*At least one live birth following ovarian stimulation

^†^Adjusted for previous segmentation, previous live birth, and whether this was the last cycle

## Discussion

In this large population cohort, we demonstrate that segmentation of the IVF cycle (i.e. freezing all embryos rather than performing an initial fresh embryo transfer and subsequent frozen embryo transfers if unsuccessful) is associated with a lower cumulative live birth rate from all embryo transfers. We showed this in a between-women analysis, with a large cohort, after adjusting for age, cycle number, and number of oocytes retrieved after ovarian stimulation. Furthermore, we observed the same lower live birth rates in segmented cycles in a within-women analysis, which controls for potential confounding by measured and unmeasured characteristics that do not change much between repeated cycles. Notably, this potentially detrimental effect of segmentation on cumulative live birth rates was only observed in the multivariable analyses. In the unadjusted analyses, the live birth rate was higher for segmented cycles, in accordance with previous registry data examining the outcome of a single fresh or frozen embryo transfer. Segmented cycles were associated with an increased risk of macrosomia and a large-for-gestational-age infant in both analyses, with these findings persisting when analyses were restricted to singleton live births and also to live births following single embryo transfer. Our findings are important for infertile couples undergoing IVF, clinicians, and policymakers, as they suggest that the increasing promotion of segmented treatment cycles [3] may be premature and unlikely to deliver what is being promised with regard to both increasing the likelihood of live birth and reducing the adverse perinatal outcomes.

Our analyses examined the cumulative live birth rate within an IVF cycle, incorporating all embryo transfers rather than just the first, which is the key outcome of interest to infertile couples. Most previous studies have only compared the transfer of fresh or frozen embryos and extrapolated their results to support segmentation [4, 10–13]. A single observational study (14,331 women) concluded that cumulative live birth rates were similar to fresh transfer among high responders but were detrimental in normal and suboptimal responders as defined by oocyte yield of 10–15 and < 10, respectively [9]. The largest RCT to date (1508 women), which did compare cumulative live birth rate within an IVF cycle, did not find an improvement in cumulative live birth rates [11], with the other two large RCTs similarly not showing a benefit if the initial embryo transfer was frozen as compared to a fresh transfer or a reduced time to pregnancy with segmentation [13, 23]. Specifically, Chen and colleagues reported a cumulative live birth rate of 62.3% for the initial frozen embryo transfer and 59.7% for the fresh embryo group (rate ratio 1.04, 95% CI 0.96 to 1.13) [11], with the subsequent trial in women without PCOS the live birth rates for the initial frozen embryo group and the fresh embryo group of 48.7% and 50.2%, respectively (rate ratio 0.97, 95% CI 0.89 to 1.06; *P* = 0.50) [23]. At 12 months of follow-up, Vuong and colleagues reported a cumulative live birth of 48.8% in the frozen embryo group and 47.3% in the fresh embryo group (risk ratio 1.03, 95% CI 0.89 to 1.19; *P* = 0.72) [13].

That there was a negative effect of segmentation in the adjusted analyses after accounting for the important patient prognostic criteria of age, number of IVF cycles, cause of infertility, and ovarian response guide may help explain our discordance with previous superiority for frozen embryo transfer when simple fresh versus frozen analyses of registry data have been undertaken. Younger women are more likely to have more oocytes retrieved, develop blastocysts, have spare good-quality embryos available for freezing, and have euploid embryos. There would be disproportionate over-representation of any or all of these characteristics when simply comparing fresh versus frozen embryo transfers. Even in older women, those with a better ovarian response and higher oocyte yield are more likely to have frozen embryos available for transfer and better quality embryos, such that even in age-stratified results, frozen embryo groups will continue to appear to do better. Our observed detrimental effect for segmentation in unselected women, and also in our oocyte yield-stratified analysis, may reflect the partial loss of viable embryos during cryopreservation. All freezing techniques are associated with the loss of some embryos, but this adverse effect is known to be greatest for slow freezing and freezing at the earlier stages of embryo development. For these clinical practices which dominated at the time of this study cleavage-stage survival rates were reported to be 60–91% [24], with recent improvements in laboratory techniques, our observed differences may be attenuated. Our results may also highlight that optimal endometrial preparation regimes have yet to be elucidated [25]. A mixture of natural and medicated frozen embryo transfer cycles with variable degrees of luteal support may contribute to the poorer results with segmentation. Replication of our analyses in patients solely exposed to extended culture and blastocyst vitrification will be of interest and help clarify the generalisability of our findings.

The observed reduced risk of low birthweight and SGA in segmented cycles is consistent with previous studies [4, 26]. This was not accompanied by a reduced risk of prematurity, with similar results when we restricted analyses to singleton births, possibly due to a detrimental effect of ovarian stimulation on placentation, which is temporally overcome with segmentation [4]. The biological plausibility for an impact on placentation is supported by recent observations that both high oestradiol at the end of ovarian stimulation [27] and supraphysiological maternal oestradiol during the first trimester [28] are associated with an increased risk of SGA. Animal model data suggests that oestrogen critically regulates trophoblast invasion and exposure to excessive oestrogen in early gestation impairs spiral artery invasion—a known determinant of intrauterine growth restriction [29]. Single embryo transfer mitigated the risk of prematurity and low birthweight in non-segmented cycles, suggesting that some of the observed detrimental effects may reflect transfer of a non-viable second embryo [19], with our observations adding weight to the case for elective single embryo transfer to optimise perinatal outcomes [19].

We observed a higher risk of LGA in segmented compared to non-segmented cycles, with an absolute rate of 18% (rather than the expected 10% based on the 90th percentile threshold definition) when analyses were restricted to singleton live births or single embryo transfer. Several studies have shown that the children born after the transfer of frozen embryos are at an increased risk of macrosomia and large for gestational age [7, 30], with the most recent analysis of siblings suggesting a causal contribution of freezing to increased birthweight [31]. The mechanism however remains unclear, with both epigenetic modifications during freezing and thawing and the different maternal endocrine and endometrial environment proposed [31]. We did not observe any difference in the risk when comparing perinatal outcomes after frozen embryo transfer only, suggesting that the increased risk of LGA is a result of only frozen embryo transfers taking place in a segmented cycle. Given the well-established risks of large for gestational age on obstetric and long-term offspring outcomes [32], along with no strong evidence for increased live birth rates, this would caution against the widespread adoption of segmentation.

We appreciate that segmentation as it is currently understood may not appear to be equivalent to the medically indicated cancelled fresh cycles, which will have dominated at the time of the study. However, elective cryopreservation of all embryos was historically principally undertaken for the prevention of ovarian hyperstimulation syndrome (OHSS), which is the same primary indication for which segmentation would be considered today [1]. The timing of the decision to segment the cycle, the ovarian stimulation strategies, and the mode of triggering final oocyte maturation may have changed, but the principal outcome of not having a fresh transfer and all embryos being cryopreserved is identical. The further development and understanding of the potential advantages, including the concept of the endometrium being hindered by the stimulation [33], was an attempt to explain why frozen embryo transfers were associated with better clinical outcomes when registry data was compared, or as an explanation for the observed differences in the initial randomised controlled trials, it was not the primary reason for considering segmentation of the cycle.

As we do not have the indication for segmentation, alternative medical indications for fresh cycle cancellation may have contributed to the observed worse cumulative cycle outcomes, for example, poor endometrial development, which may recur in subsequent cycles. However, analyses of all endometrial thicknesses for all cycles undertaken in Canada demonstrated that 99.1% of all fresh cycles had an endometrial thickness ≥ 6 mm and 96.1% had ≥ 7 mm, suggesting that this is an infrequent finding and would not be responsible for the effect size observed [15]. Women with PCOS may be more likely to over-respond and have a medically indicated segmented cycle and are known to have worse obstetric outcomes [34]. However, fertilisation rates have been shown to be equivalent in women with PCOS [35], and with contemporary laboratory techniques, they may even be anticipated to have higher cumulative live birth rates due to their potential for a higher oocyte yield [36]. We noted that segmented cycles had more embryos created and more embryos transferred and therefore do not anticipate that our results are due to poorer prognosis patients in the segmented group. Other indications such as concerns regarding the adverse effect of progesterone elevation on the endometrium and embryo quality [37, 38] may also have contributed. However, as progesterone concentration reflect the ovarian stimulation [39], this would emphasise the need for clinicians to not disregard optimal gonadotrophin dosing simply because the OHSS risk can be mitigated.

Our study has a number of strengths: we included over 200,000 women undertaking more than 330,000 IVF cycles and 399,000 embryo transfers. We incorporated all eligible IVF cycles undertaken in the UK over a 10-year period. The HFEA is subject to parliamentary jurisdiction and the data source subject to routine quality assurance checks and performs well in terms of completeness and accuracy [40]. Through a unique maternal identifier, we linked each woman to every IVF cycle and every embryo transfer they had undertaken, irrespective of whether the woman had moved clinic. This enabled us to also identify over 3000 women who had experienced both segmented and non-segmented cycles, and complete a within-women analyses, which, consistent with analyses in all eligible women, showed lower live birth rates in segmented compared to non-segmented cycles. We also demonstrated robust findings across a range of additional analyses.

We acknowledge that our analyses had some limitations. We have utilised population data derived from a decade of treatment, which will include heterogeneous clinical and laboratory practices [18, 19, 41, 42]. However, this heterogeneity reflects contemporary global clinical practice, with units continuing to differ in freezing protocols, and with a large variation in the technical skills of embryologists. We did not have the reason for segmentation of the cycle, but we have adjusted all analyses for the underlying cause of infertility, and our results were largely unchanged. We did not have detailed information on whether vitrification or slow freezing was used or the stage of embryo development when freezing occurred. Information on the developmental stage at transfer was missing on 37% of the segmented cycles but just 1% of non-segmented cycles; therefore, we did not adjust for this in our main analyses. As a result, our main analyses could be influenced by residual confounding which would tend to bias the results towards a beneficial effect of segmentation on live birth rate (given blastocysts may better survive freezing and be more likely to result in a live birth). This confounding might therefore mask a stronger association between non-segmentation and live birth. In additional analyses, we controlled for cleavage or blastocyst stage in those participants with data on this. Because of the differential missingness, these complete case analyses might suffer from selection bias [22]. However, as long as missingness in the covariate is unrelated to the outcome, then the complete case analysis will be unbiassed—even if missingness is related to the covariate [22]. In support of this, the results were essentially the same as our main analyses.

We appreciate that there have been continued improvements in vitrification survival rates in recent years and that with current vitrification techniques, the observed differences may be attenuated. We were unable to obtain precise dates from clinics regarding the switch from slow freezing to vitrification as for many systems ran in parallel depending on which stage of embryo development was being frozen. Restricting our analyses to the most recent 2 years of available data, where there would be potentially less heterogeneity of practice and extended culture and vitrification of blastocysts may be more prevalent, did not lead to any substantial changes in our results, which further supports our main results being relevant to contemporary populations. In some countries, e.g. Germany, slow freezing still dominates due to legislative reasons, and replication of our results with contemporaneous data in other settings would be useful to confirm our conclusions.

Prior to 2009, women were only able to store embryos for 5 years in the UK, and after 2009, this was extended to 10 years with further storage possible. Consequently, we appreciate that not all women had used all their embryos, and this may have underestimated the overall cumulative live birth rate. As the number of women who had used all their embryos was higher in segmented cycles, it is likely that the overall cumulative live birth rate was more severely underestimated in non-segmented cycles compared with segmented cycles. Similarly, we are unable to accurately calculate the full reproductive potential of a single IVF cycle, the “one and done” approach as it is not clear in this population dataset the desired number of children [43] or how this may affect the decision to segment the cycle. For the protection of anonymity, we do not have the date of births for the offspring and are therefore unable to calculate the time to pregnancy for each of the two strategies, but appreciate the importance of this outcome for patients. Lastly, despite the HFEA having a legislative and regulatory requirement to collect data, the option to participate in research with the data was introduced in 2009. We appreciate that we may therefore have incomplete data capture for the later years of the study, but this would only have biassed the results if consent for data was not randomly distributed between segmented and non-segmented cycles, which is unlikely given that the Consent to Disclosure form is completed prior to commencing ovarian stimulation. The consistency of our findings when restricted to the last 3 years of cycles only (i.e. 2011–2013) with main analyses also suggests that this change has not biassed our findings. The other possibility is collider bias, where an unmeasured variable affected both research participation and the need for segmentation and a separate unmeasured variable affected both research participation and the outcomes of interest [44]. Replication of our findings in population cohorts where there is complete data capture would be useful in addressing potential bias attributable to unknown reasons for participation.

We did not have detailed data on some potential confounders, such as maternal smoking, body mass index, and socio-economic position. We did not have detailed information on embryo quality and acknowledge that if women with poorer quality embryos were more likely to freeze all embryos than those with good-quality embryos, this may influence the results and explain why segmentation was associated with worse outcomes. However, this is unlikely as freezing is generally restricted to good-quality embryos, and our within-women analysis reached the same conclusion as our between-women analysis for live birth success, suggesting that the effect of unmeasured confounders that are likely to change little within women is limited. The within-women analysis, by necessity, was restricted to women who had more than one ovarian stimulation cycle, and we were unable to examine the perinatal outcomes due to the small number (155) of women who had a live birth from a segmented cycle and a live birth from a non-segmented cycle. We did not have data on endometrial development; however, large population studies have suggested that endometrial thicknesses < 6 mm affects < 1% of cycles [15]. Lastly, we did not have data on whether embryos were replaced in a medicated or natural cycle, with no data on the nature or extent of luteal support [25].

We acknowledge that segmentation of the cycle is beneficial for ovarian hyperstimulation syndrome (OHSS) prevention, but we were unable to undertake analyses separately in women with and without a diagnosis of OHSS, as information on this diagnosis is not accurately recorded within the HFEA dataset and the indication for segmentation was not available. However, this is unlikely to have a major impact on our findings as the incidence of OHSS is low (1% of cycles) [45], and inclusion of these women would tend to bias the findings towards a benefit of segmentation in the general IVF patient population of both those with and without OHSS. We also appreciate that preimplantation genetic testing (PGT) of blastocysts will require segmentation of the cycle. However, PGT has not been universally adopted, pending the outcome of an international multicentre trial (NCT02268786) [46].

## Conclusion

Cryopreservation is an essential aspect of assisted conception, and its widespread application has maximised the safety and efficacy of treatment. Our findings show that 30 years after the introduction of cryopreservation, elective freezing of all embryos for all patients if used in an unselected manner, without heed of laboratory expertise or the patient profile, may potentially compromise the cumulative live birth rate within a single IVF cycle. These findings support restricting segmentation to patients where there is a clear clinical need, such as the prevention of ovarian hyperstimulation or preimplantation genetic testing.

## Supplementary information

**Additional file 1 **: **Figure S1**. Risk ratios of perinatal outcomes following first live-birth within a cycle, for segmented cycles compared with non-segmented cycles, in 82,561 singleton live-births from 202,968 women undergoing 337,148 cycles of IVF. * adjusted. **Figure S2**. Risk ratios of perinatal outcomes following first live-birth within a cycle, for segmented cycles compared with non-segmented cycles, in 18,685 live-births as a result of single embryo transfer from 202,968 women undergoing 337,148 cycles of IVF. Very preterm birth and very low birth weight are not shown due to fewer than 8 incidences in segmented cycles. **Figure S3**. Live-birth rate ratios for segmented cycles compared with non-segmented cycles, in 81,682 cycles of IVF between 1 January 2011 and 31 December 2012. **FigureS4**. Risk ratios of perinatal outcomes following first live-birth within a cycle, for segmented cycles compared with non-segmented cycles, in 26,094 live-births from 81,682 cycles of IVF between 1 January 2011 and 31 December 2012. **Figure S5**. Live-birth rate ratios for segmented cycles compared with non-segmented cycles, stratified according to stage of embryo in 329,621 cycles of IVF. **Figure S6**. Live-birth rate ratios from the first embryo transfer (fresh in segmented cycles compared with frozen in non-segmented cycles), in 202,968 women undergoing 337,148 cycles of IVF. **Figure S7**. Risk ratios of perinatal outcomes following live-birth from the first embryo transfer (fresh in segmented cycles compared with frozen in non-segmented cycles), in 96,098 live-births from 202,968 women undergoing 337,148 cycles of IVF. **Figure S8**. Risk ratios of perinatal outcomes following first live-birth within a cycle, excluding live-birth following a fresh embryo transfer, for segmented cycles compared with non-segmented cycles, in 10,928 live-births from 202,968 women undergoing 337,148 cycles of IVF.

## Data Availability

The data that support the findings of this study are available from HFEA (https://www.hfea.gov.uk/about-us/our-data/), and restrictions apply to the availability of these data, which were used under approval for the current study, and so are not publicly available.

## Abbreviations

DI: Donor insemination
GIFT: Gamete intrafallopian transfer
HFEA: Human Fertilisation and Embryology Authority
IVF: In vitro fertilisation
LGA: Large for gestational age
LBW: Low birthweight
OHSS: Ovarian hyperstimulation syndrome
RCT: Randomised controlled trial
SGA: Small for gestational age
ZIFT: Zygote intrafallopian transfer
