# Supplementary material for: Live birth rates and perinatal outcomes when all embryos are frozen compared with conventional fresh and frozen embryo transfer: a cohort study of 337,148 in vitro fertilisation cycles
Source: BMC Med. 2019 Nov 13;17:202. doi: 10.1186/s12916-019-1429-z (PMC6852977; doi:10.1186/s12916-019-1429-z)
Supplement: Supplementary file 1 — Additional file 1 : Figure S1. Risk ratios of perinatal outcomes following first live-birth within a cycle, for segmented cycles compared with non-segmented cycles, in 82,561 singleton live-births from 202,968 women undergoing 337,148 cycles of IVF. * adjusted. Figure S2. Risk ratios of perinatal outcomes following first live-birth within a cycle, for segmented cycles compared with non-segmented cycles, in 18,685 live-births as a result of single embryo transfer from 202,968 women undergoing 337,148 cycles of IVF. Very preterm birth and very low birth weight are not shown due to fewer than 8 incidences in segmented cycles. Figure S3. Live-birth rate ratios for segmented cycles compared with non-segmented cycles, in 81,682 cycles of IVF between 1 January 2011 and 31 December 2012. FigureS4. Risk ratios of perinatal outcomes following first live-birth within a cycle, for segmented cycles compared with non-segmented cycles, in 26,094 live-births from 81,682 cycles of IVF between 1 January 2011 and 31 December 2012. Figure S5. Live-birth rate ratios for segmented cycles compared with non-segmented cycles, stratified according to stage of embryo in 329,621 cycles of IVF. Figure S6. Live-birth rate ratios from the first embryo transfer (fresh in segmented cycles compared with frozen in non-segmented cycles), in 202,968 women undergoing 337,148 cycles of IVF. Figure S7. Risk ratios of perinatal outcomes following live-birth from the first embryo transfer (fresh in segmented cycles compared with frozen in non-segmented cycles), in 96,098 live-births from 202,968 women undergoing 337,148 cycles of IVF. Figure S8. Risk ratios of perinatal outcomes following first live-birth within a cycle, excluding live-birth following a fresh embryo transfer, for segmented cycles compared with non-segmented cycles, in 10,928 live-births from 202,968 women undergoing 337,148 cycles of IVF. [file 12916_2019_1429_MOESM1_ESM.pdf]

Web extra material

Supplemental statistical methods

We adjusted for observed confounders by including them as categorical variables in the generalized linear models. The categories were: for age: less than 35 years, 35-39 years, and greater than 39 years at the time of ovarian stimulation; for cycle number: first cycles and later cycles; for number of oocytes retrieved: greater than 15 oocytes or less than or equal to 15 oocytes. These thresholds are based on previous evidence, for example live-birth rates notable decline after age 35 years, and increase up to retrieval of 15 oocytes by thereafter change little

Supplementary Figure 1

Risk ratios of perinatal outcomes following first live-birth within a cycle, for segmented cycles compared with non-segmented cycles, in 82,561 singleton live-births from 202,968 women undergoing 337,148 cycles of IVF.

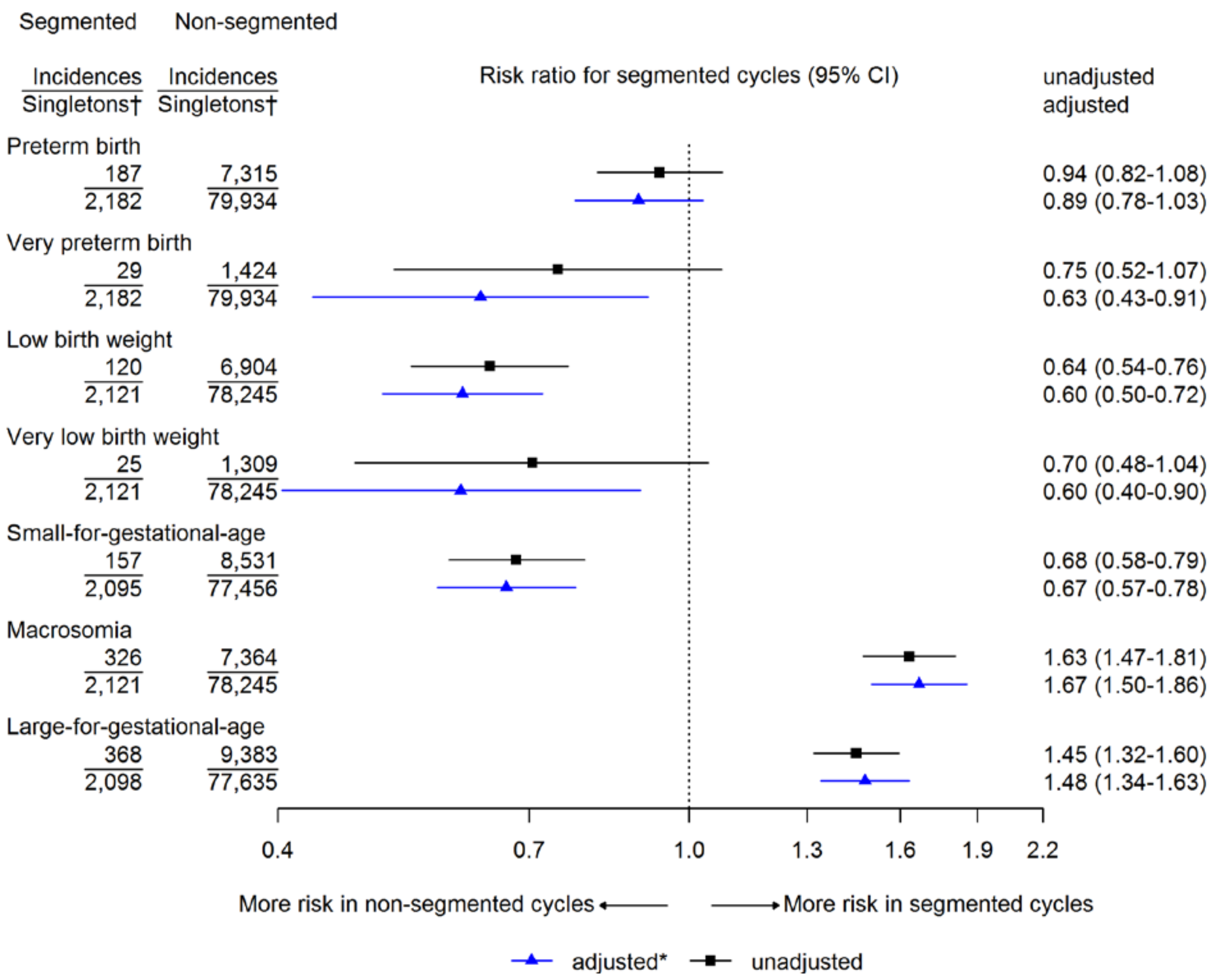

* adjusted for age, cycle number, oocytes retrieved, cause of infertility and previous IVF pregnancy

† where information is non-missing

Supplementary Figure 2

Risk ratios of perinatal outcomes following first live-birth within a cycle, for segmented cycles compared with non-segmented cycles, in 18,685 live-births as a result of single embryo transfer from 202,968 women undergoing 337,148 cycles of IVF. Very preterm birth and very low birth weight are not shown due to fewer than 8 incidences in segmented cycles.

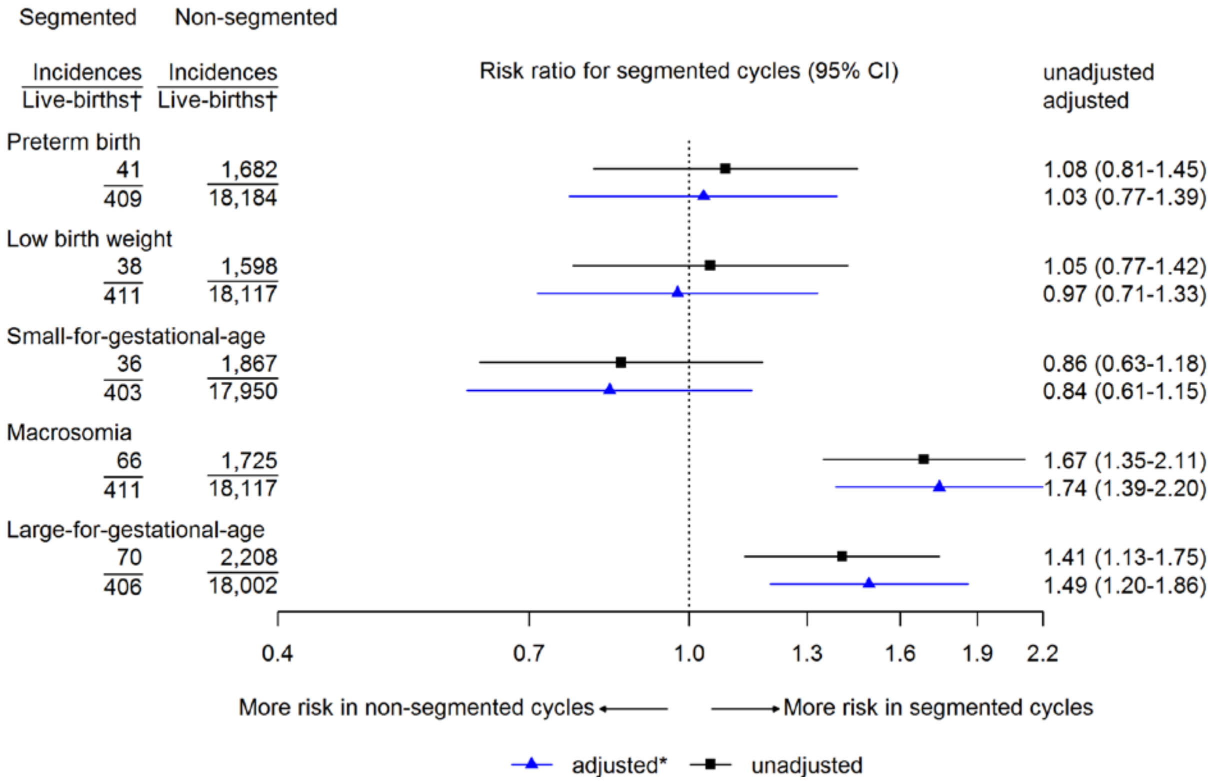

* adjusted for age, cycle number, cause of infertility, oocytes retrieved, and previous IVF pregnancy

† where information is non-missing

Supplementary Figure 3

Live-birth rate ratios for segmented cycles compared with non-segmented cycles, in 81,682 cycles of IVF between 1 January 2011 and 31 December 2012.

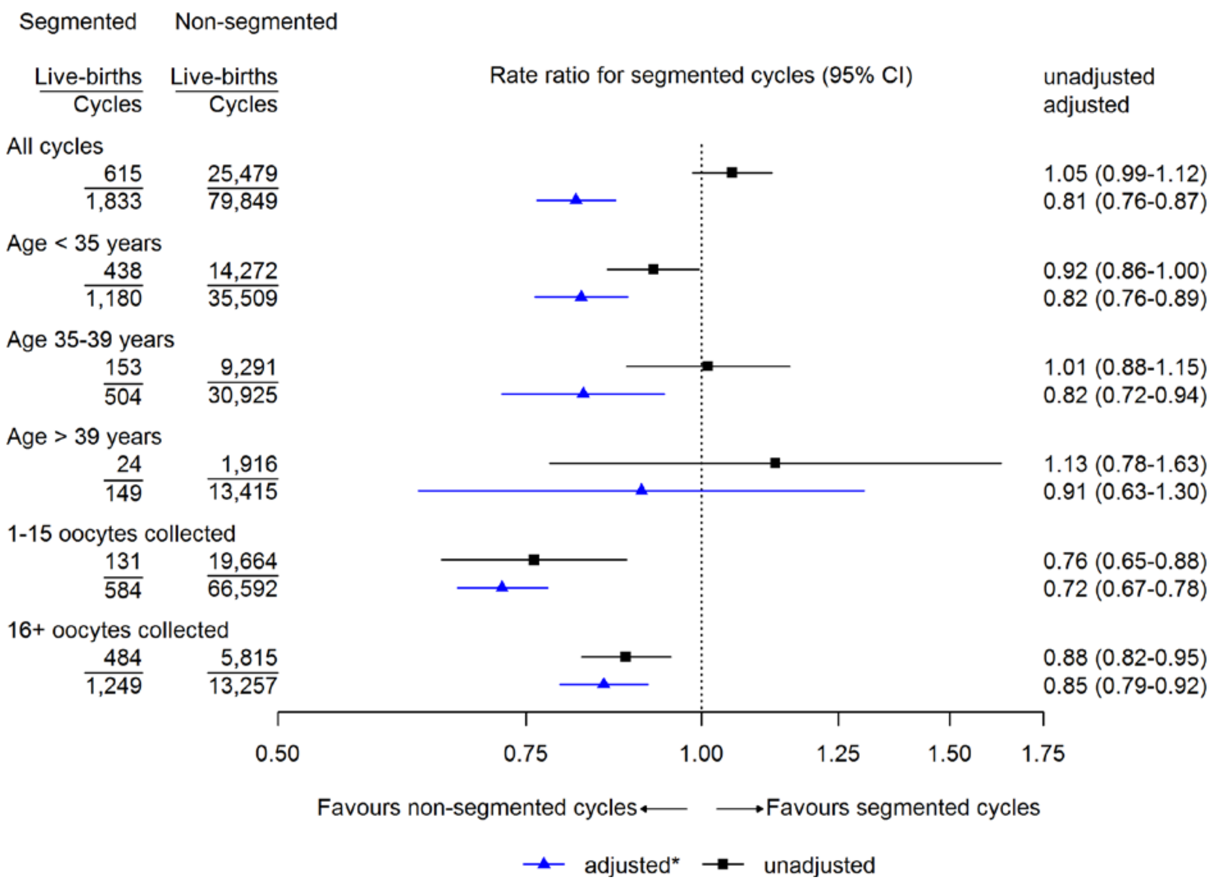

* adjusted for age (where not stratified), cycle number, cause of infertility and oocytes retrieved (where not stratified)

Supplementary Figure 4

Risk ratios of perinatal outcomes following first live-birth within a cycle, for segmented cycles compared with non-segmented cycles, in 26,094 live-births from 81,682 cycles of IVF between 1 January 2011 and 31 December 2012.

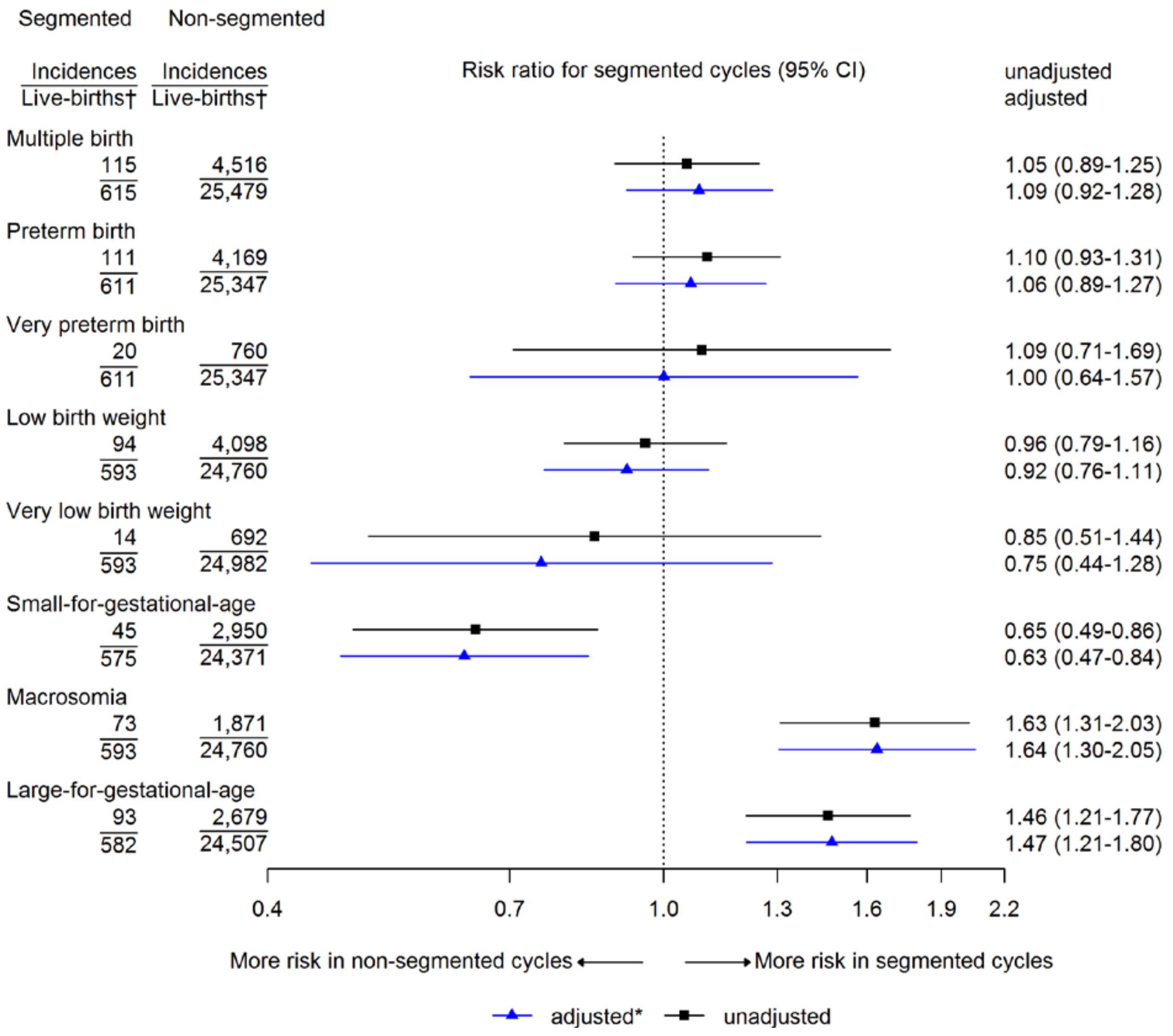

* adjusted for age, cycle number, cause of infertility and oocytes retrieved

† where information is non-missing

Supplementary Figure 5

Live-birth rate ratios for segmented cycles compared with non-segmented cycles, stratified according to stage of embryo in 329,621 cycles of IVF.

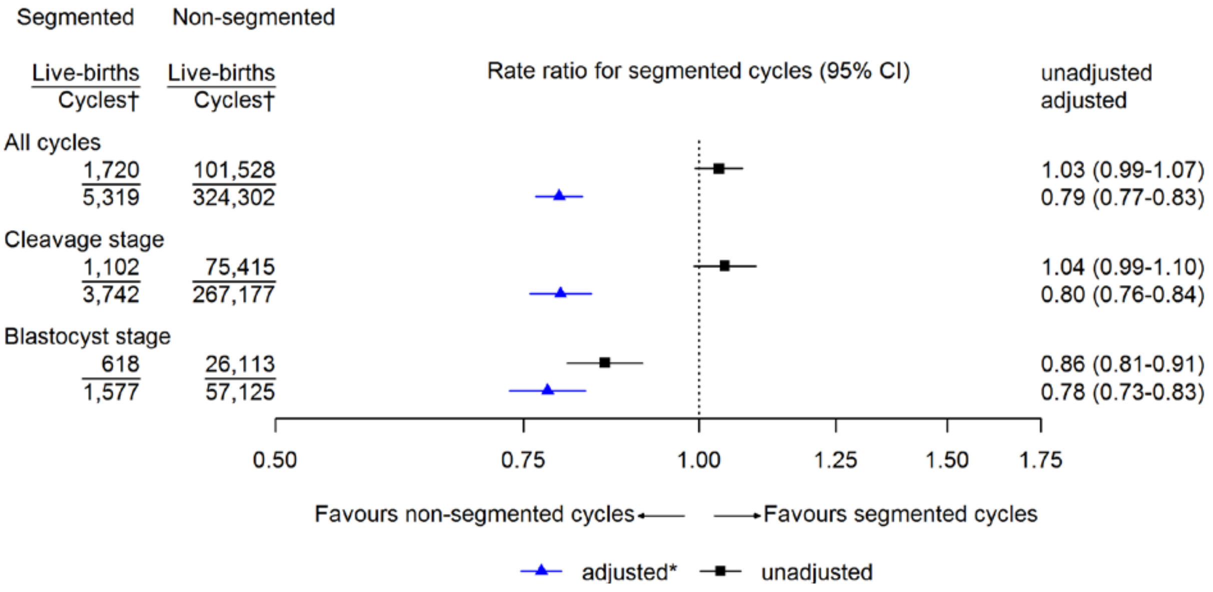

* adjusted for age, cycle number, cause of infertility, oocytes retrieved and stage of embryo transfer (where not stratified)

† where information is non-missing

Supplementary Figure 6

Live-birth rate ratios from the first embryo transfer (fresh in segmented cycles compared with frozen in non-segmented cycles), in 202,968 women undergoing 337,148 cycles of IVF.

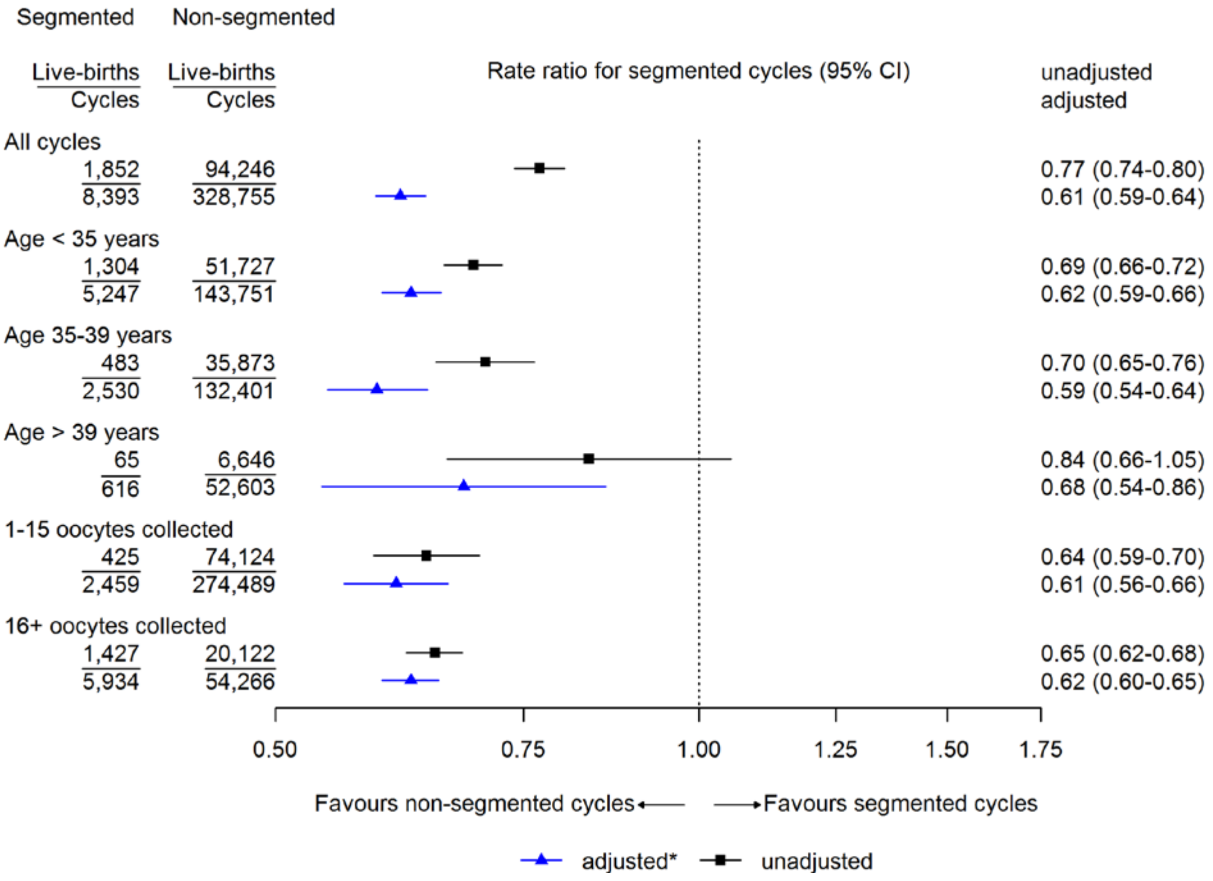

* adjusted for age (where not stratified), cause of infertility, cycle number and oocytes retrieved (where not stratified)

Supplementary Figure 7

Risk ratios of perinatal outcomes following live-birth from the first embryo transfer (fresh in segmented cycles compared with frozen in non-segmented cycles), in 96,098 live-births from 202,968 women undergoing 337,148 cycles of IVF.

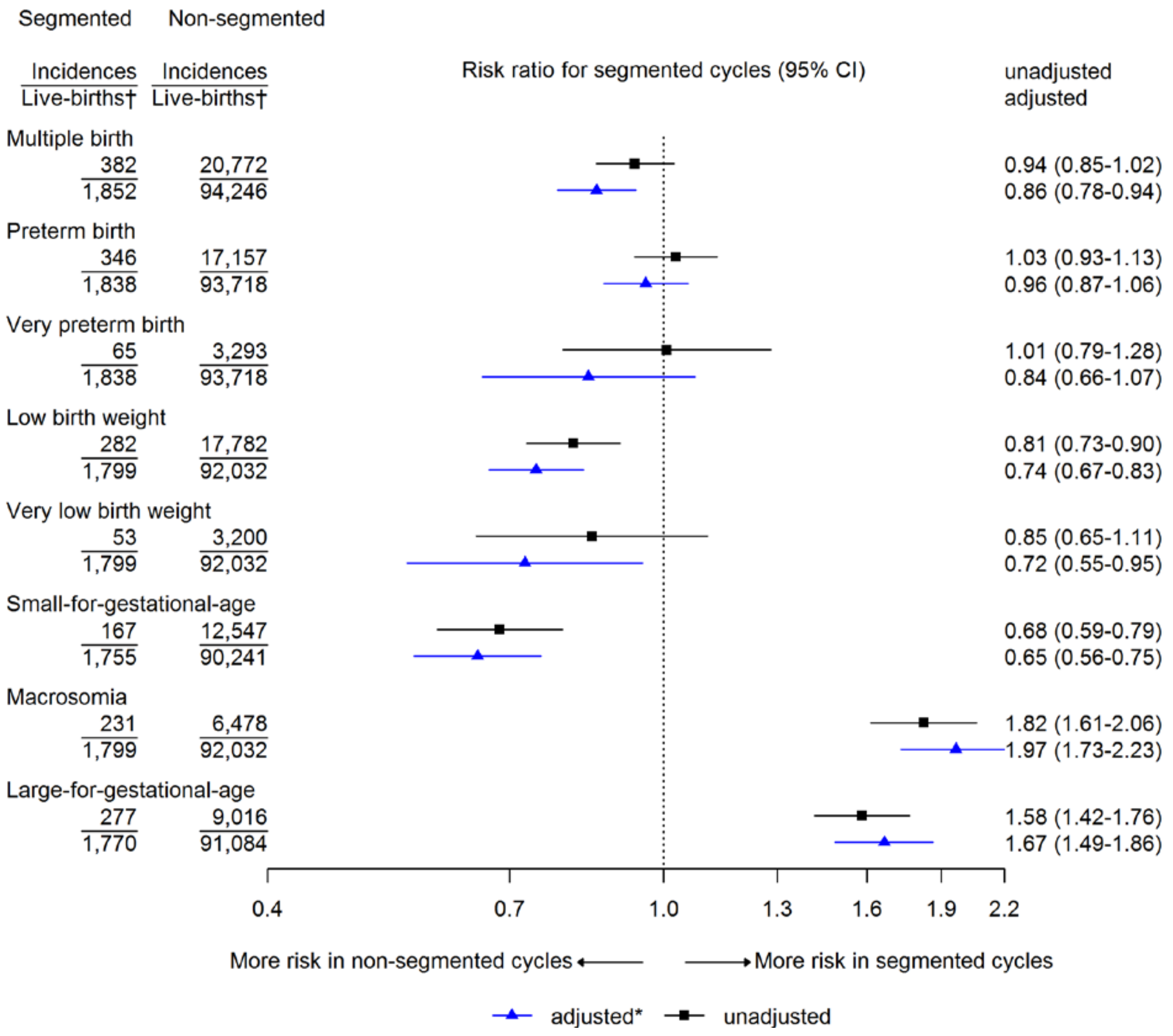

* adjusted for age, cycle number, cause of infertility and oocytes retrieved

† where information is non-missing

Supplementary Figure 8

Risk ratios of perinatal outcomes following first live-birth within a cycle, excluding live-birth following a fresh embryo transfer, for segmented cycles compared with non-segmented cycles, in 10,928 live-births from 202,968 women undergoing 337,148 cycles of IVF.

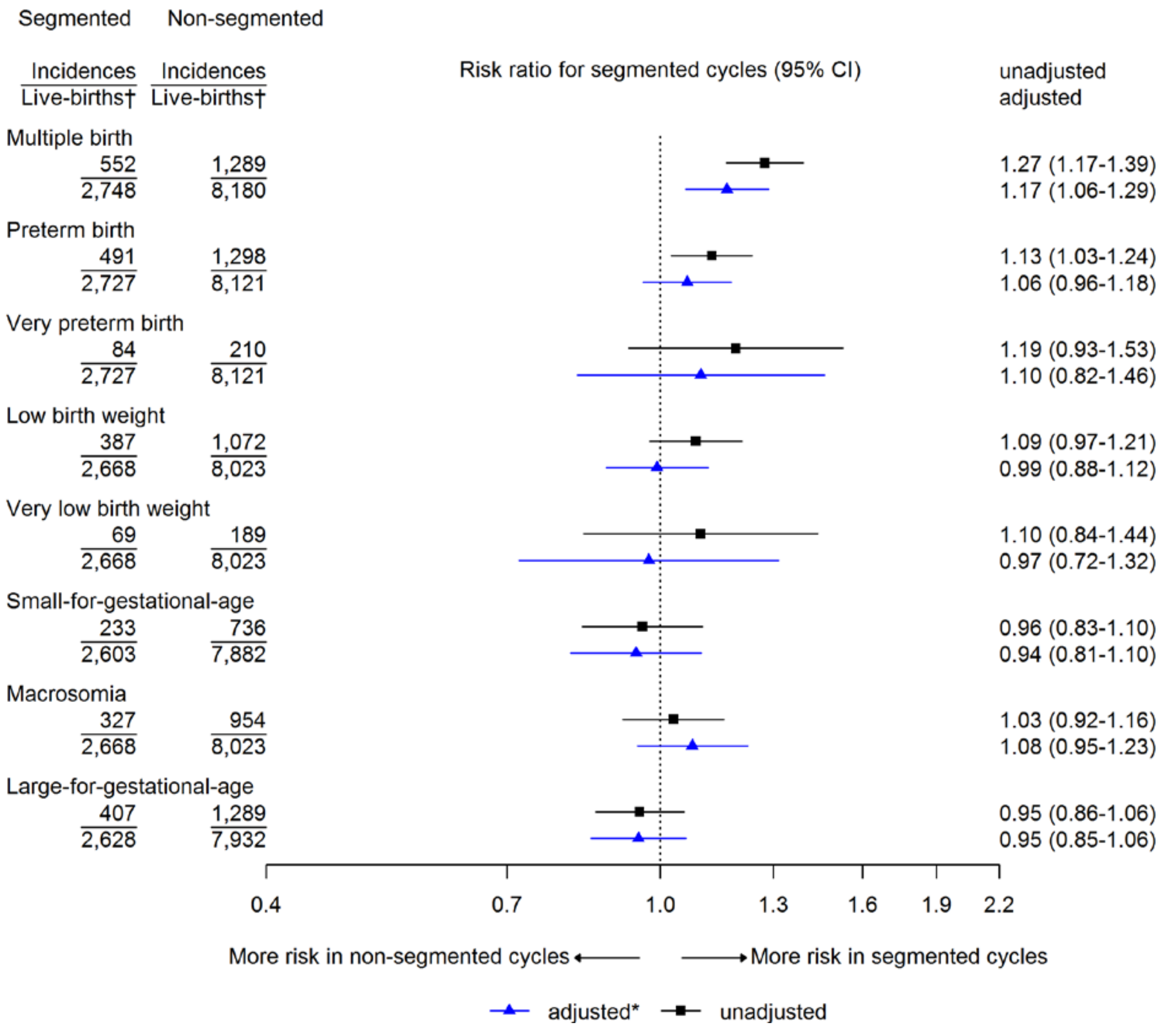

* adjusted for age, cycle number, cause of infertility and oocytes retrieved

† where information is non-missing
